# Potassium channels of T lymphocytes take center stage in the fight against cancer

**DOI:** 10.1186/s40425-016-0202-5

**Published:** 2017-01-17

**Authors:** Laura Conforti

**Affiliations:** Department of Internal Medicine, Division of Nephrology, University of Cincinnati, Cincinnati, OH USA

**Keywords:** Potassium ions, Tumor microenvironment, Immunotherapy

## Abstract

A recent study by Eil at al. published in Nature in September 2016 provides evidence that alterations of the K^+^ homeostasis of tumor infiltrating lymphocytes (TILs) in necrotic areas of the tumor microenvironment (TME) suppress the function of effector T cells. Furthermore, they establish that overexpression of K^+^ channels in T lymphocytes counterbalances this negative effect of the TME and restores the ability of TILs to function, ultimately leading to increased survival of tumor bearing mice. Thus, K^+^ channels in T lymphocytes become interesting new targets for novel immunotherapies in cancer. This Commentary discusses Eil’s finding in the context of the central role that K^+^ channels play in the suppressed state of TILs as they mediate the immunosuppressive effects of multiple conditions of the TME including hypoxia and adenosine.

Immunotherapies are revolutionizing the way cancer is treated and they have shown remarkable advances in treatment outcomes. The efficacy of immunotherapy, such as immune checkpoint inhibitors, in cancer relies on the ability of the therapy to augment the cytolytic activity/functionality of tumor-specific T cells, increase their migration into the tumor, and maintain their functionality in the immunosuppressive tumor microenvironment (TME) [1]. While a high number of cytotoxic and helper Th1 T cells in the tumors is often reported to be of good prognostic value, other features such as their location and functional state within the tumor determine their ability to eradicate cancer cells. Unfortunately, in various solid tumors, tumor infiltrating T lymphocytes (TILs) exhibit multiple functional defects including reduced proliferation, cytotoxicity and cytokine production (IL-2 and IFNγ) and increased cell death [1, 2].

Various features of the TME have been implicated in the reduced functionality of TILs. Solid tumors implement a series of complementary mechanisms that are hostile to the functionality of effector T cells. These include: disabling the antigen presentation machinery (like downregulating MHC class I molecules), upregulating surface ligands that drive T cell exhaustion and fostering a milieu that is enriched in immunosuppressive factors [1]. Rapidly dividing tumor cells create areas of low oxygen tension (hypoxia) and necrosis which are associated with poor prognosis [3, 4].

In a recent article by Eil et al., which appeared in Nature in September 2016, the authors reported a novel mechanism by which necrosis in solid tumors interferes with T cell function [4]. They showed that the death of cancer cells in necrotic areas leads to release of potassium ions (K^+^) and their accumulation in the extracellular compartment at concentrations 5–10 times higher than normal serum levels. Exposure of T lymphocytes to such high concentrations of K^+^ inhibits the transcription of genes mediating the activation response of T cells to antigen presentation and, ultimately, effector functions such as IFNγ and IL-2 release. Eil et al. also discovered the mechanism underlying this phenomenon: excessive extracellular K^+^ results in an increase in intracellular K^+^ concentration that ultimately leads to the blockade of the T cell receptor (TCR) activated Akt/mTOR signaling pathway via the phosphatase PP2A. In accordance with the causative effects of suppressing the Akt/mTOR pathway, high extracellular K^+^ inhibited nutrient consumption and polarization of resting CD4^+^ T cells into effector cells, while promoting the development of immunosuppressive regulatory T cells (T_reg_). Importantly, in this paper the authors showed that an ionic imbalance contributes to TIL dysfunction in cancer.

Maintaining the appropriate distribution of ions across the cell membrane is essential for the function of all cell types. In T lymphocytes, ion channels, transporters and pumps are the “master switches” that work in concert to maintain the physiological distribution of ions (gradients) at the cell quiescent resting state and to allow the rapid redistribution of ions upon encounter of an antigen which drives TCR signaling and associated functional responses [5]. In Eil’s paper the authors reported that the accumulation of intracellular K^+^ in T lymphocytes in the presence of the excessive extracellular K^+^ is due to an imbalance between the K^+^ entry into the cell (through a pump, the Na^+^, K^+^-ATPase) and the efflux of K^+^ through K^+^ channels.

In human T lymphocytes K^+^ efflux is controlled by two K^+^ channels: Kv1.3 (a voltage-dependent K^+^ channel activated by membrane depolarization) and KCa3.1 (a K^+^ channel activated by a rise in cytosolic Ca^2+^; also known as IK1 or Gardos channel). These channels control the membrane potential (the voltage difference across the cell membrane arising from differences in ions’ distribution) and are known to work in concert with Ca^2+^ channels to control the TCR-mediated Ca^2+^ influx necessary for NF-AT mediated T cell activation [5]. This phenomenon has been well described and indeed blockade of Kv1.3 and KCa3.1 channels suppresses T cell function. Eil and colleagues proposed a novel additional mechanism by which Kv1.3 and KCa3.1 channels contribute to the reduced functionality of TILs in necrotic areas of tumors. They proposed that reduced function of K^+^ channels contributes to the accumulation of K^+^ into the cells which ultimately downregulates Akt/mTOR signaling pathway; an effect that is independent on changes in membrane potential and intracellular Ca^2+^ levels. The authors also found that overexpression of K^+^ channels provides the K^+^ efflux that can restore the intracellular K^+^ concentration of T cells to physiological levels and correct Akt/mTOR signaling and functional defects. Importantly, they reported that overexpression of Kv1.3 channels restored the anti-tumor functionality of TILs and, ultimately, reduced tumor burden and increased survival in tumor-bearing mice [4]. Similar outcomes were produced by overexpression or pharmacological activation of KCa3.1 channels. These findings underscore the importance of K^+^ channels in tumor clearance and their therapeutic potentials.

Overexpression of K^+^ channels can have multiple beneficial effects in the functionality of TILs as it also counteract the immunosuppressive function of other elements of the TME which signal, in part, through K^+^ channels. Upstream to necrosis, hypoxia and adenosine contribute to the failure of immune surveillance in cancer [3]. Areas of severe hypoxia generate because of the abnormal vasculature that forms in the tumor (with blind ends and leaky vessels) and the excess consumption of oxygen by proliferative cancer cells. Adenosine, a purine nucleoside produced by tumor cells under hypoxia and by T_reg_ accumulating in the tumor, can reach concentrations in solid tumors 100 fold higher than those of normal tissues. Both hypoxia and adenosine limit T cell function, and indeed Sitkovsky’s group has elegantly shown that correcting these by treating tumor-bearing mice with respiratory hyperoxia enhances T cell cytotoxicity and cytokine release, improves tumor regression and the efficacy of immunotherapies (adoptive T cell transfer and dual blockade of CTLA-4 and PD-1) [6]. Hypoxia and adenosine inhibit Kv1.3 and KCa3.1 channels, respectively, in T lymphocytes thus limiting Ca^2+^-mediated cell functions such as proliferation, cytokine release and motility [7, 8]. Indeed TILs freshly isolated from human tumors have been shown to present with low TCR-mediated Ca^2+^ fluxes that limit their ability to fight cancer cells [2, 9]. Still, TILs were able to recover a Ca^2+^ response to TCR stimulation in culture, suggesting that disruption of the TME is sufficient to restore their functionality [9]. Recently, our laboratory has reported that the defect in Ca^2+^ fluxes of cytotoxic TILs in head and neck cancer patients is due to reduced Kv1.3 expression which correlates with reduced proliferative and cytotoxic capacities of TILs [10]. These findings position Kv1.3 channels as markers of functionally competent cytotoxic T cells and further strengthen the therapeutic potentials of targeting K^+^ channels of T lymphocytes in cancer. Overall, multiple immunosuppressive inputs of the TME converge on K^+^ channels in T lymphocytes making them attractive targets for novel combination immunotherapies.

## Abbreviations

TME: Tumor microenvironment
Th1: Type 1 helper T cells
TILs: Tumor infiltrating lymphocytes
IL-2: Interleukin 2
IFNγ: Interferon gamma
MHC: Major histocompatibility complex
K^+^: Potassium ions
TCR: T cell receptor
Akt: AKT serine/threonine kinase also known as protein kinase B or PKB
mTOR: Mammalian target of rapamycin
PP2A: Protein phosphatase 2A
T_reg_: Regulatory T cells
Kv1.3: Voltage-gated potassium channel encoded in humans by the KCNA3 gene
KCa3.1: Calcium-activated potassium channel encoded in humans by the KCNN4 gene, also known as IK1 or Gardos channel
NF-AT: Nuclear factor of activated T cells
Ca^2+^: Calcium ions
CTLA-4: Cytotoxic T-lymphocyte associated protein 4
PD-1: Programmed cell death protein 1
